# Microbubble enhanced mass transfer efficiency of CO_2_ capture utilizing aqueous triethanolamine for enzymatic resorcinol carboxylation

**DOI:** 10.1039/d0ra08690h

**Published:** 2021-01-20

**Authors:** Daniel Ohde, Benjamin Thomas, Simon Matthes, Shunya Tanaka, Paul Bubenheim, Koichi Terasaka, Michael Schlüter, Andreas Liese

**Affiliations:** Institute of Technical Biocatalysis, Hamburg University of Technology Hamburg Germany liese@tuhh.de; Institute of Multiphase Flows, Hamburg University of Technology Hamburg Germany; Department of Applied Chemistry, Keio University Yokohama Japan; School of Science for Open and Environmental Systems, Graduate School of Science and Technology, Keio University Yokohama Japan

## Abstract

The present study focuses on the aeration of aqueous triethanolamine acting as reaction medium for biocatalytic carboxylations. For enhancing mass transfer in a bubble column reactor, microbubble aeration is applied and compared to conventional macrobubble aeration. Application of a 0.5 μm porous sparger enables microbubble CO_2_ aeration with bubble size distributions below 150 μm in Sauter mean diameter, correlating with the highest measured mass transfer rates. During CO_2_ saturation of the aqueous triethanolamine, bubble size distributions changed according to the level of CO_2_ saturation. For microbubbles, less foaming was observed compared to macrobubble aeration by a 10 μm porous sparger. This microbubble effect is attributed to their accelerated dissolution assisted by the Laplace pressure lowering the amount of bubbles reaching the surface of the liquid. The experiments reveal that the rate of interfacial area generation, which is calculated based on measured bubble size distributions, influences the biocatalyst activity.

## Abstract 2

An inline method to investigate microbubble enhanced mass transfer and its effect on a model biocatalyst utilizing CO_2_ for carboxylation.

## Graphical abstract

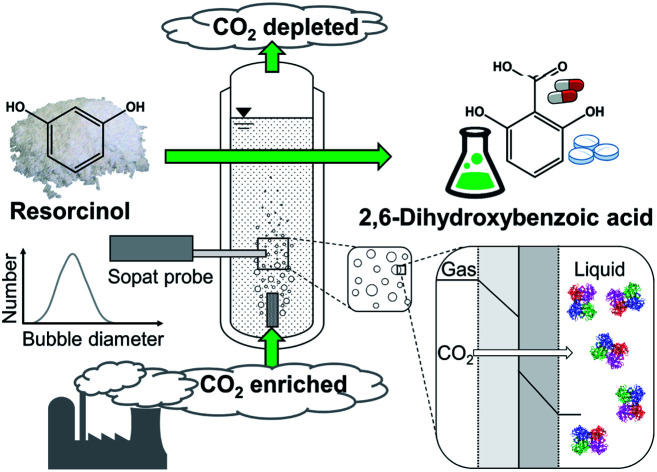

## Introduction

**Fig. 1:**
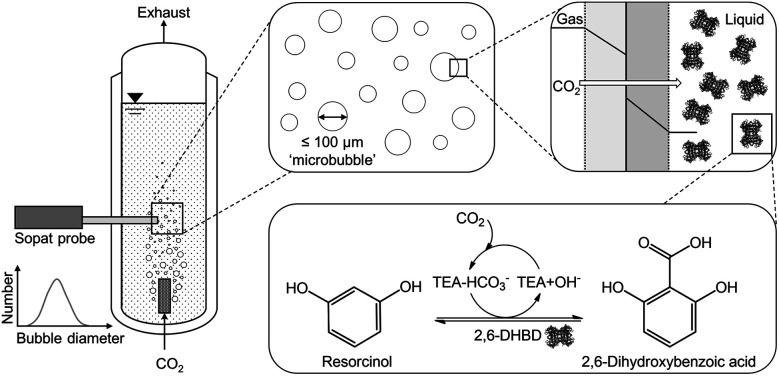
Enzymatic carboxylation of resorcinol in a bubble column with microbubble aeration for CO_2_ mass transfer. TEA = triethanolamine; 2,6-DHBD = 2,6-dihydroxybenzoic acid decarboxylase; two phase system with indicated boundary layer, dotted lines.

CO_2_ capture based on chemical absorption is the most mature industrial scale technology to reduce CO_2_ emission.^1^ Exhaust gases and exhaled air are treated with solvents and especially amines, on which the majority of the research is focused on, to bind CO_2_. The major challenge of this technology is the high energy requirement for the solvent regeneration, which is performed by heating it up to 120 °C.^1^ Beside the high energy consumption for the regeneration, further energy is required for the aeration. Additionally, finding a suitable storage location for the CO_2_, degradation of primary and secondary amines by oxygen as well as the formation of acid need to be addressed.^2^ Currently, using geological formations for storage is the most advanced approach.^3^ However, other applications of the sequestrated CO_2_ are being researched to enable it as a resource.^4,5^ For this approach, biocatalysis could be crucial as the application of carbonic anhydrase was already shown to improve the mass transfer significantly resulting in an overall cost reduction of the CO_2_ capture process.^6^ Further studies involving biocatalysis in CO_2_ loaded amines proved that the bound CO_2_ can be used in the biocatalytic carboxylation of phenolic compounds to produce valuable aromatic acids.^7^ For this reaction system, a separate CO_2_ loading has to be performed to neutralize aqueous amine solutions due to their alkaline nature. Otherwise, the alkaline condition deactivates the biocatalyst utilized in this process as they are only stable in a limited pH range with the pH optimum being at 8.5.^8^ Primary and secondary amines first form carbamates which then produce carbonates in water, whereas tertiary amines produce carbonates directly *via* a base catalyzed mechanism without carbamate formation.^9,10^ In contrast to using carbonate salts as CO_2_ source, gaseous CO_2_ is used in the amine-mediated carboxylation (Fig. 1).

Previous studies showed that higher amine concentrations improved the achievable yield in the thermodynamic limited biotransformation as well as reduced the produced bubble sizes when using membrane spargers.^11^ Even microbubble aeration, which is defined as aeration with bubbles smaller than 100 μm in diameter,^12^ was achieved. Utilizing microbubbles provides a high volume specific surface area as well as shrinking behavior coupled with accelerated dissolution assisted by the Laplace pressure to enhance the mass transfer.^13,14^ Especially the Laplace pressure accelerates the dissolution due to the increasing curvature of the boundary layer of shrinking of microbubbles.^15^ This highly efficient aeration technique has the potential in reducing the necessary CO_2_ gas stream as well as the waste of unreacted CO_2_.

In this contribution, we report on the mass transfer performance of the CO_2_ loading of aqueous triethanolamine (TEA) as integral part for the utilization in biocatalytic carboxylation. Furthermore, the effect of microbubble aeration on the biocatalyst, 2,6-dihydroxybenzoic acid decarboxylase (2,6-DHBD) from *Rhizobium* sp., catalyzing the carboxylation of resorcinol, is investigated. The reaction product 2,6-dihydroxybenzoic acid is important in a wide range of industrial, pharmaceutical and agricultural applications.^16^ Our results provide new insights into the CO_2_-amine system and help promoting the applicability of this system for industry.

## Experimental

### General

All chemicals were obtained from Sigma-Aldrich (Darmstadt, Germany), except triethanolamine, which was purchased from Carl Roth (Karlsruhe, Germany). Carbon dioxide 4.5 (≥99.995%) was obtained from Linde (Pullach, Germany). For the aeration in the bubble column experiments, 1′′ long and ½′′ in diameter stainless steel filters with 2 μm and 10 μm pores were purchased from Techlab (Braunschweig, Germany) and a 0.5 μm PerfectPeak® solvent inlet filter from Adaptas (Massachusetts, USA) were used as sparger. The pH measurements were performed with a Knick (Berlin, Germany) pH-Meter 766 Calimatic.

### Dynamic *k*_L_*a* measurements

Dynamic *k*_L_*a* measurements were performed in a double jacket bubble column with nitrogen and air gassing method.^17^ The 360 mm long double jacket column had an inner diameter of 27 mm. For oxygen detection, PreSens (Regensburg, Germany) oxygen sensor SP-PSt3-NAU-D10-YOP was used. Measurements were performed in 150 ml 1 M TEA at 30 °C reaching a fill level of 225 mm. For the linearization of the data, eqn (1) was used with the oxygen concentration at saturation c* (mg ml^−1^) and measured oxygen concentrations in the liquid *c*_l_ (mg ml^−1^) at the different time points *t* (s).1
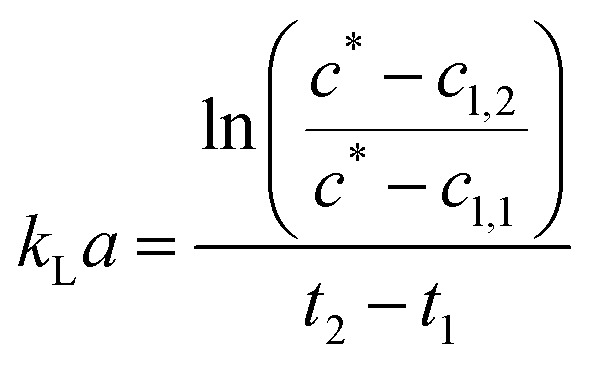


### Bubble size distribution

Measurements of bubble sizes were performed with a Sopat-VI Sc probe from Sopat (Berlin, Germany) in a bubble column setup. The column had an inner diameter of 31.9 mm and was used with a working volume of 150 ml. The probe was centered 36 mm above the sparger. The measuring range with the Sopat Sc is 9–1200 μm. The gap on top of the Sopat probe was adjusted to 2 mm for the 0.5 μm sparger and between 5–10 mm for the 2 μm and 10 μm sparger with smaller gaps at larger TEA concentrations. For the bubble size distribution (BSD) measurement in unsaturated aqueous TEA (during CO_2_ loading at a CO_2_ gassing rate of 100 ml min^−1^), the elapsed time between start of the CO_2_ aeration and initialization of the Sopat measurement was ensured to be below 30 seconds. Nevertheless, the initially unsaturated aqueous TEA solution is further loaded with CO_2_ during the Sopat measurement itself making it only possible to get an approximation of the BSD in unsaturated aqueous TEA. For each BSD, 200 images at a rate of 5 images per seconds were recorded, totaling a measuring time of 40 seconds. The obtained images were analyzed by the integrated image analysis software. Lognormal regressions were performed with the data analysis and graphing software Origin 2019b from OriginLab (Massachusetts, USA).

### Enzymatic reactions

Production and purification of the 2,6-DHBD were performed according to previously reported protocols.^5^ Carboxylation reactions were carried out in the double jacket bubble column with an inner diameter of 27 mm and a working volume of 150 ml. The reactions were started by enzyme addition to the previously CO_2_ saturated 1 M TEA solution containing the substrate resorcinol.

### Sampling procedure and RP-HPLC method

Reaction samples were diluted in trifluoroacetic acid to stop the reaction and remove amine-bound CO_2_. Samples were mixed and centrifuged for 5 min at 13 000 rpm. Supernatant was injected in an Agilent (Waldbronn, Germany) LC-1100 HPLC system equipped with a diode array detector and a LichroCART 250-4 Lichrospher 100 RP column (5 μm). The separation was performed according to the previously reported protocol.^11^ Typical retention times were 7.0 min for resorcinol and 8.3 min for 2,6-dihydroxybenzoic acid.

## Results and discussion

### Effects of CO_2_ saturation on the aeration

**Fig. 2:**
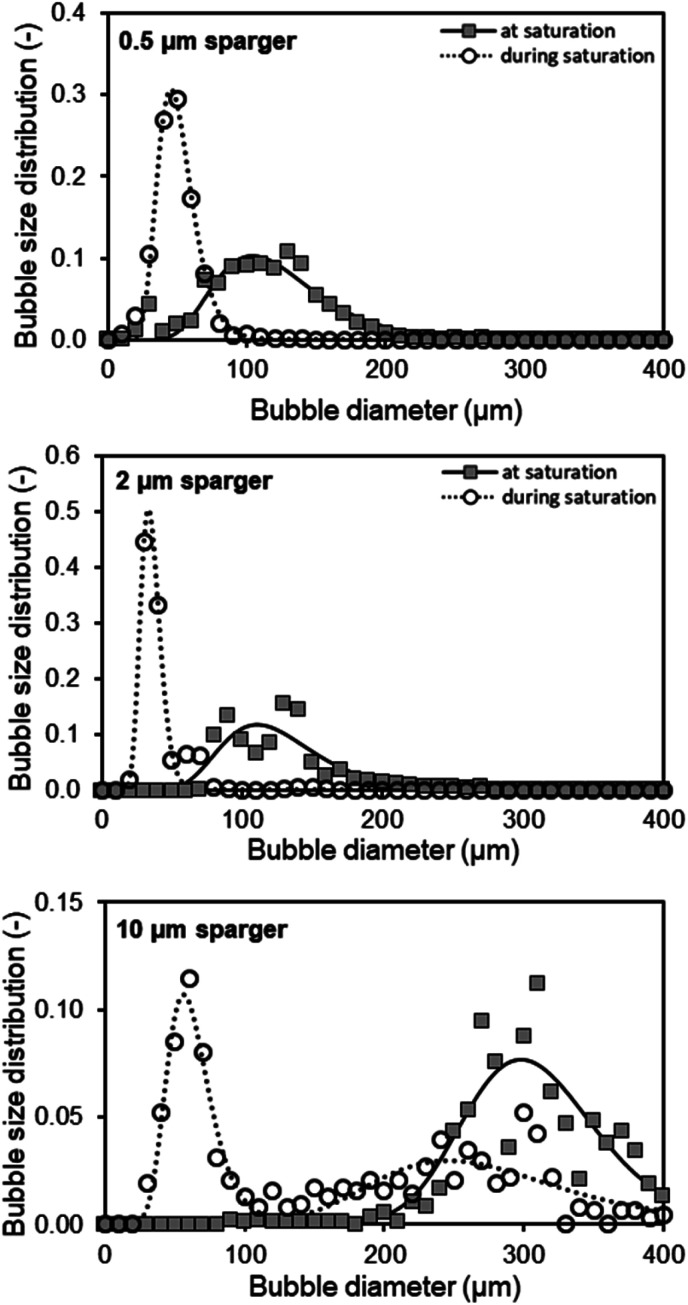
CO_2_ bubble size distributions during initial saturation and at saturation of 150 ml 1 M triethanolamine with 100 ml min^−1^ using different spargers in a bubble column with an inner diameter of 31.9 mm under ambient conditions. The bubbles were detected with the Sopat-VI Sc probe-based microscope and analysed by the integrated image analysis software. Log-normal regressions with the following *R*^2^ for each at and before CO_2_ saturation were calculated, respectively: 0.5 μm sparger: 0.92 and 0.99; 2 μm sparger: 0.81 and 0.98; 10 μm sparger: 0.84 and 0.70 (bimodal).

During CO_2_ saturation of an aqueous 1 M TEA solution at 30 °C, the pH shifts from initially 10.8 to 7.5. Besides the changing pH, it is observed that for the initial aeration, sparse or no foaming occurs, whereas increased foaming takes place when the CO_2_ saturation of TEA is reached. For quantification of this observed foaming behavior, the BSD is measured with CO_2_ during initial loading (in the first 30 seconds) of unsaturated TEA as well as after reaching the maximum saturation, indicated by a stable pH. Three spargers with identical geometric dimensions and different mean pore diameters of 0.5, 2 and 10 μm are characterized in a bubble column setup at 30 °C (Fig. 2).

For these spargers, the BSDs shift to larger bubbles after CO_2_ saturation of 1 M TEA with a gas flow of 100 ml min^−1^ CO_2_. Both, the 0.5 μm and 2 μm sparger, produce similar BSDs (Fig. 2). However, the 10 μm sparger shows a deviation with a peculiar distribution. During saturation, the initial bimodal distribution changes to an unimodal one, whereas both submillibubble peaks are in the same 300 μm size range (Fig. 2). This could indicate that the initially produced submillibubbles shrink to microbubbles due to the mass transport during saturation. Therefore, the submillibubble peak shifts slightly from 300 μm to approximately 240 μm. Reaching in part the microbubble region as intermediate step, the mass transfer gets enhanced by the Laplace pressure. This leads to the formation of the characteristic microbubble peak. In contrast to the bimodal distribution of the 10 μm sparger, both the 0.5 μm and 2 μm sparger already produce bubbles in the 100 μm range at saturation, whereby only unimodal distributions are produced. Characteristic shrinking of bubbles in the micrometer range was observed by Tanaka *et al.* for the dissolution of air in water at different air saturation levels by microbubble aeration.^13^ In this study on single freely rising microbubbles, it was demonstrated that the mass transfer coefficient *k*_L_ increases for shrinking microbubbles, which agrees with the Ranz and Marshall's correlation.^13^ Therefore, the enhanced complete dissolution of microbubbles during their slow rise to the surface results in less foaming as less bubbles reach the surface.

In contrast to experiments with single bubbles, coalescence needs to be considered at aeration with high bubble concentrations independent of bubble size. The decreasing bubble diameter in the BSD for undersaturated aqueous TEA solutions would mean that effects that increase the bubble size such as coalescence, play a minor role in the initial saturation process compared to the accelerated dissolution assisted by the Laplace pressure. This hypothesis is supported by the decreasing zeta potential of smaller bubble sizes,^18^ which enhances the repulsion of the negatively charged surfaces and diminishes coalescence of microbubbles.^19^ Even though coalescence enhances break-up of foam, this effect can only occur when bubbles reach the liquid surface. Reaching the CO_2_ saturation of TEA, the pH is lowered in consequence from an alkaline to a neutral value, which increases the zeta potential of microbubbles.^18^ Takahashi *et al.* demonstrated that the zeta potential of microbubbles changes from −110 mV above pH 10 to positive values below pH 4.5. Considering the changing pH of 1 M aqueous TEA from 10.8 to 7.5, coalescence enhances and contributes to the increasing bubble diameters of the measured BSD (Fig. 2). Coalescence behavior of microbubbles and the existence of the bimodal distribution in Fig. 2 (10 μm sparger) further agrees with the microbubble study of Matthes *et al.*, in which similar effects were observed. They measured increasing Sauter mean diameter and the appearance of bimodal BSDs nearing the top of the reactor.^20^ As the result of this coalescence behavior, emerging submillibubbles have higher rising velocities, much shorter residence times and show accelerated dissolution assisted by Laplace pressure only near the microbubble region.

### Mass balance simulation

The theoretical rising velocity *v* of micro- and macrobubbles can be calculated following the classical approach using the Stokes law (eqn (2)).^21^ This incorporates the bubble diameter (*d*_B_), gravitational constant (*g*), densities of the gas (*ρ*_g_) and liquid (*ρ*_l_) as well as the dynamic viscosity (*η*).2
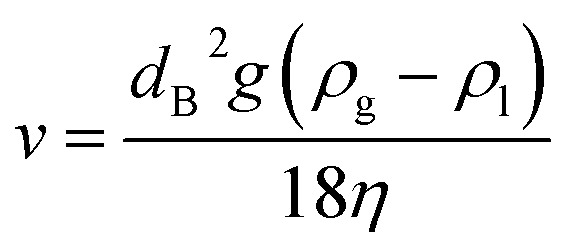


In case of microbubbles, it was demonstrated that the Hadamard–Rybczynski interpretation of Naiver–Stokes, which takes the boundary condition, the internal viscosity and the dispersive phase into account, matches experimental results closer.^22–24^ According to the work of Iwakiri *et al.* (2017), mass balance simulations of different sized bubbles are performed in the aqueous TEA system.^15^ This is used for further investigation of the hypothesis, that the initially produced submillibubbles in Fig. 2 shrink to microbubbles due to mass transport. The simulation and physical properties for aeration in 1 M aqueous TEA are summarized in Table 1. For the preparation of aqueous TEA solutions, vigorous mixing is required to homogeneously dissolve TEA in water saturating the solution with air oxygen and nitrogen in the process. Therefore, as starting point for the simulation, an initial saturation of the aqueous TEA solution with air is assumed as no further degassing was performed. Here, we define the saturation ratio *S* as3
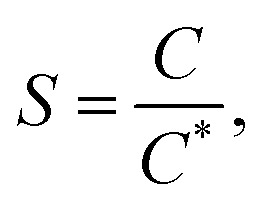
where *C* is the dissolved gas concentration and C* is the maximum solubility of the gas. Therefore, air saturated solutions reach *S*_air_ = 1 and CO_2_ saturated solutions reach *S*_CO_2__ = 1.

**Table 1:** Simulation parameter and physical properties in 1 M aqueous TEA

Simulation parameter	Value	Literature^25–29^
Reactor height *h* [m]	0.152	
Atmospheric pressure *P*_atm_ [Pa]	1.013 × 10^5^	
Temperature *T* [K]	303.15	
Surface tension *σ* [N m^−1^]	62.8 × 10^−3^	Vázquez *et al.*, 1996
Dynamic viscosity *η* [Pa s]	1.2659 × 10^−3^	Ko *et al.*, 2001
Liquid density *ρ*_l_ [kg m^−3^]	1018.2	Ko *et al.*, 2001
Henry's constant *H*(CO_2_)^a^ [mol m^−3^ Pa^−1^]	3.46 × 10^−4^	Danckwerts, 1966
Henry's constant *H*(O_2_)^a^ [mol m^−3^ Pa^−1^]	1.2 × 10^−5^	Sander, 2015
Henry's constant *H*(N_2_)^a^ [mol m^−3^ Pa^−1^]	6.4 × 10^−6^	Sander, 2015
Diffusion coefficient *D*(CO_2_)^a^ [m^2^ s^−1^]	1.14 × 10^−9^	Ko *et al.*, 2001
Diffusion coefficient *D*(O_2_)^a^ [m^2^ s^−1^]	2.2 × 10^−9^	Himmelblau, 1964
Diffusion coefficient *D*(N_2_)^a^ [m^2^ s^−1^]	2.0 × 10^−9^	Himmelblau, 1964

a Values for gas properties in pure water.

**Fig. 3:**
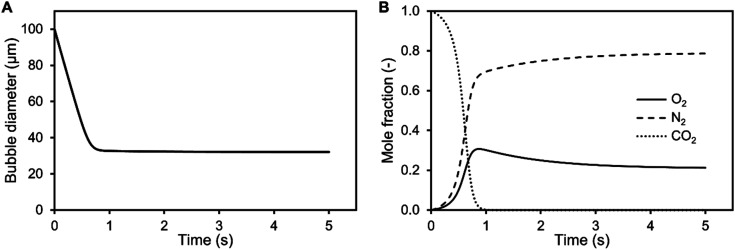
Shrinking of a 100 μm CO_2_ bubble (A) and change in the inner bubble mole fraction (B) during aeration in 1 M aqueous triethanolamine at 30 °C and 101.3 kPa simulated with Matlab. The initial saturation of the solution was set to *S*_air_ = 1 and *S*_CO_2__ = 0.

It is shown from simulation that the bubble size decreases during aeration of air saturated aqueous TEA for a 100 μm CO_2_ bubble (Fig. 3). During the shrinking, the bubble gas volume is rapidly exchanged and equilibrated with air oxygen and nitrogen (Fig. 3B) stabilizing the bubble. This prevents further rapid shrinking. Nonetheless, the bubble is calculated to completely dissolve after 29.1 s due to accelerated dissolution assisted by Laplace pressure. During the process of dissolution, it is calculated that the single bubble rises approximately 8.5 mm. Therefore, it completely dissolves before reaching the surface. Only CO_2_ bubbles above 540 μm would reach the surface during the saturation process (*S*_air_ = 1). After CO_2_ saturation of 1 M aqueous TEA (*S*_CO_2__ = 1), CO_2_ bubbles above 140 μm are expected to reach the surface before dissolution. This supports the observation of increased foaming when reaching CO_2_ saturation in the bubble column experiments. Taking the determined BSD from Fig. 2 into account, it is clear that a significant amount of bubbles is bigger than 140 μm and would reach the surface producing the observed foam.

**Fig. 4:**
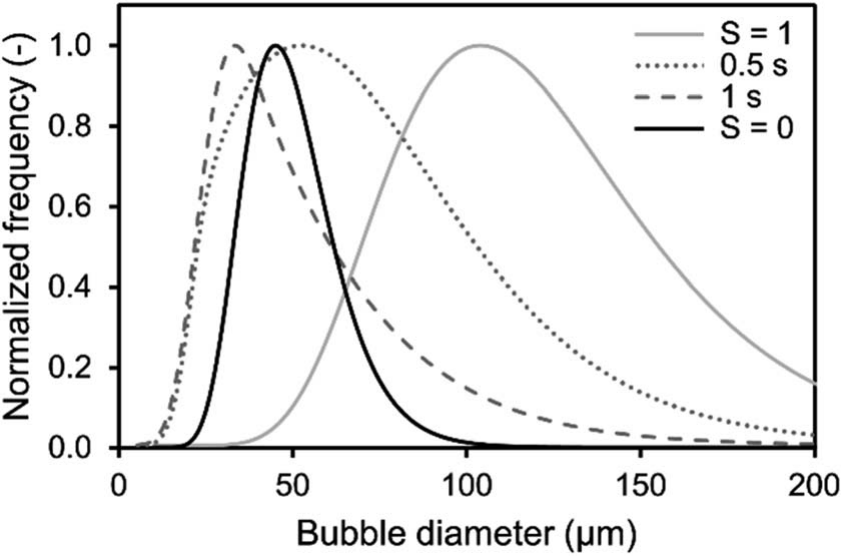
Comparison of simulated and obtained lognormal CO_2_ bubble size distribution from experiments during initial saturation (*S* = 0) and at CO_2_ saturation (*S* = 1) of 1 M aqueous triethanolamine using a 0.5 μm sparger from (Fig. 2). The simulated distributions are calculated using a mass transfer model of single free rising bubbles. The distribution changes according to the passed time after bubble formation, which is shown for 0.5 s and 1 s. The frequency is normalized to the same peak height of the distributions.

Additionally, the determined BSD at CO_2_ saturation is used to predict the theoretical BSD, which would be measured during the initial saturation of air saturated aqueous TEA. The corresponding simulation is performed exemplarily for the aeration experiments utilizing the 0.5 μm sparger (Fig. 4). The BSD in Fig. 4 are normalized to the same peak height of the distributions. It is shown that the passed time, after starting the aeration, is influencing the BSD significantly. The rapid shrinking shifts the BSD to smaller bubbles. This demonstrates the challenge of measuring the initial BSD. Already, the measurement with the SOPAT probe takes 0.5 s to obtain 200 pictures. In this short time frame, the BSD changes significantly as shown in Fig. 4. This can be reduced by limiting the amount of pictures further. However, the biggest influence is the initialization period of the measurement with a time delay between start of the aeration and the waiting time for the formation of a uniform multiphase flow. Nevertheless, the experimental BSD is in rough agreement with a hypothetical BSD between the 0.5 s and 1 s simulated BSD. At the same time, the width of the BSD narrows, which is consistent between experimental data and the simulation. An explanation is the previously shown counter diffusion of air nitrogen and oxygen from the liquid in the CO_2_ bubbles during their ascent to the surface.

The residual difference between the simulated and experimental BSDs is possibly caused by two major reasons: (1) errors in the initial BSD and (2) neglection of additional effects in the simulated BSD. Regarding the measurement error of the initial BSDs generated from the sparger, we only assume that it is the BSD for *S*_CO_2__ = 1 in the simulation. Although this BSD is considered to be a sufficient representative of the reality, some differences from the lognormal distribution are observed in the experimental data as shown in Fig. 2. Therefore, the error of the initial BSD may also affect the final simulation results, resulting in a difference between experiment and simulation. The exact acquisition of the initial BSDs is an issue to be considered in the future. Additional effects are neglected in the BSD simulation, such as the difference in the measurement positions of the BSDs observed by the SOPAT probe and the BSDs observed in the simulation. The SOPAT probe acquires pictures of the bubbles 31.9 mm above the sparger, whereas the simulation shows the fate of all the bubbles in the initial BSD. In other words, a mixture of bubbles of various lifetimes are photographed with the SOPAT probe, whereas the simulation captures changes in bubbles of identical lifetimes. Furthermore, the performed simulations do not take into account the liquid flow and the variation of residence time for each bubble diameter in the flow field. Additionally, effects of coalescence need to be considered when investigating rising bubble swarms. These differences between simulation and reality are also points that lead to differences in the BSD.

### Mass transfer investigation

The increasing bubble diameter in the BSD during TEA aeration with CO_2_ is lowering the volumetric mass transfer coefficient *k*_L_*a* due to the resulting decrease of the specific surface area and respective mass transfer coefficient *k*_L_ when reaching microbubbles smaller than 40 μm.^13^ Consequently, not only the CO_2_ concentration gradient during aeration of the liquid, but also the *k*_L_*a* diminishes and reduces the overall mass transfer efficiency. In an optimal process, the aeration to saturate the amine should be fast and highly efficient. For a better understanding of the mechanism, the mass transfer in aqueous TEA is investigated with the intention to achieve a more efficient aeration with small bubbles, resulting in high interfacial areas and high mass transfer rates.

Measurement of CO_2_ transfer rates in aqueous TEA solutions with a CO_2_ sensor spot SP-CD1 from PreSens (Regensburg, Germany) were carried out and resulted in no measureable CO_2_ signal. However, CO_2_ was measureable in pure water acting as control. The capable measuring range of the CO_2_ sensor is from 10 to 250 hPa *p*CO_2_. It is likely that CO_2_ was not detectable, because of the adsorption and hydration caused by the amine. In general, CO_2_ measurements in aqueous amine solutions are complex due to the CO_2_ equilibrium with the amine^10^ in addition to the hydrated CO_2_ equilibria.^30^ Therefore, the established approach of estimating the CO_2_*k*_L_*a* on basis of measured air oxygen *k*_L_*a* and comparison of the diffusion constants (*D*_L_) is conducted (eqn (4)).^31^4
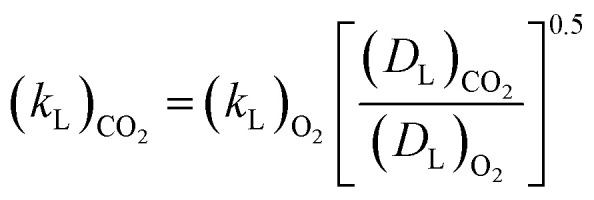


**Fig. 5:**
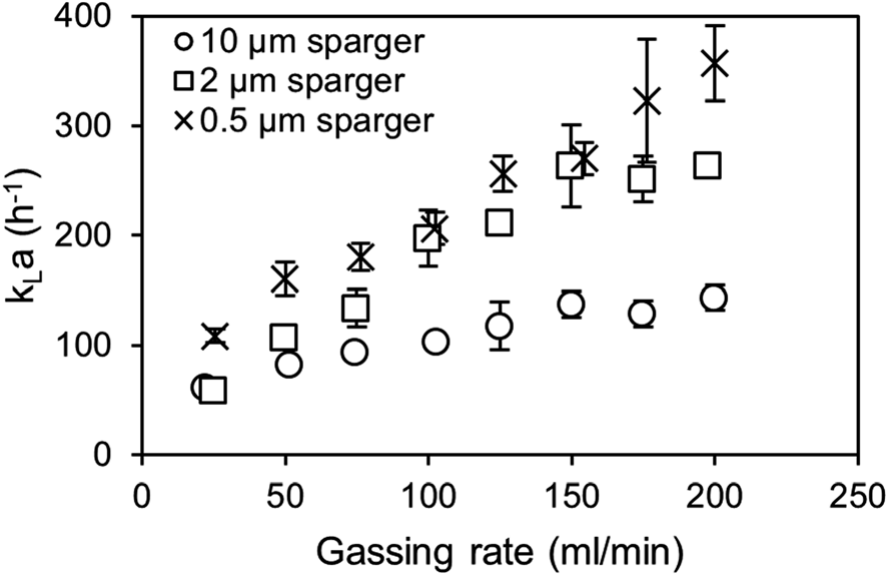
Dynamic air oxygen *k*_L_*a* measurements of air with different spargers in 1 M triethanolamine at 30 °C.

Eqn (3) provides a correction factor based on the diffusion constants. This factor can be used to estimate the CO_2_*k*_L_*a* provided that the specific surface area (*a*) between the air and CO_2_ aeration is comparable.^31,32^ Therefore, the volumetric mass transfer coefficients (*k*_L_*a*) are determined for spargers with mean pore sizes of 0.5, 2 and 10 μm (Fig. 5). Regarding the air oxygen *k*_L_*a*, the 0.5 μm and 2 μm sparger perform again similarly, while utilization of the 0.5 μm sparger achieves overall slightly higher *k*_L_*a* values. From previous studies, it is known that the 0.5 μm sparger produces slightly smaller bubbles with *d*_50_ of 79 μm compared to 109 μm for the 2 μm sparger in 1 M TEA at 20 ml min^−1^ CO_2_ in a bubble column setup.^11^ The same effect is observed in the measurement with a flow rate of 100 ml min^−1^. Contrary to that, the utilization of the 10 μm sparger results in a much lower *k*_L_*a*, which can be explained by formation of bigger bubbles due to lower pore size dependent capillary pressure that need to be overcome for bubble growth.^33^

**Fig. 6:**
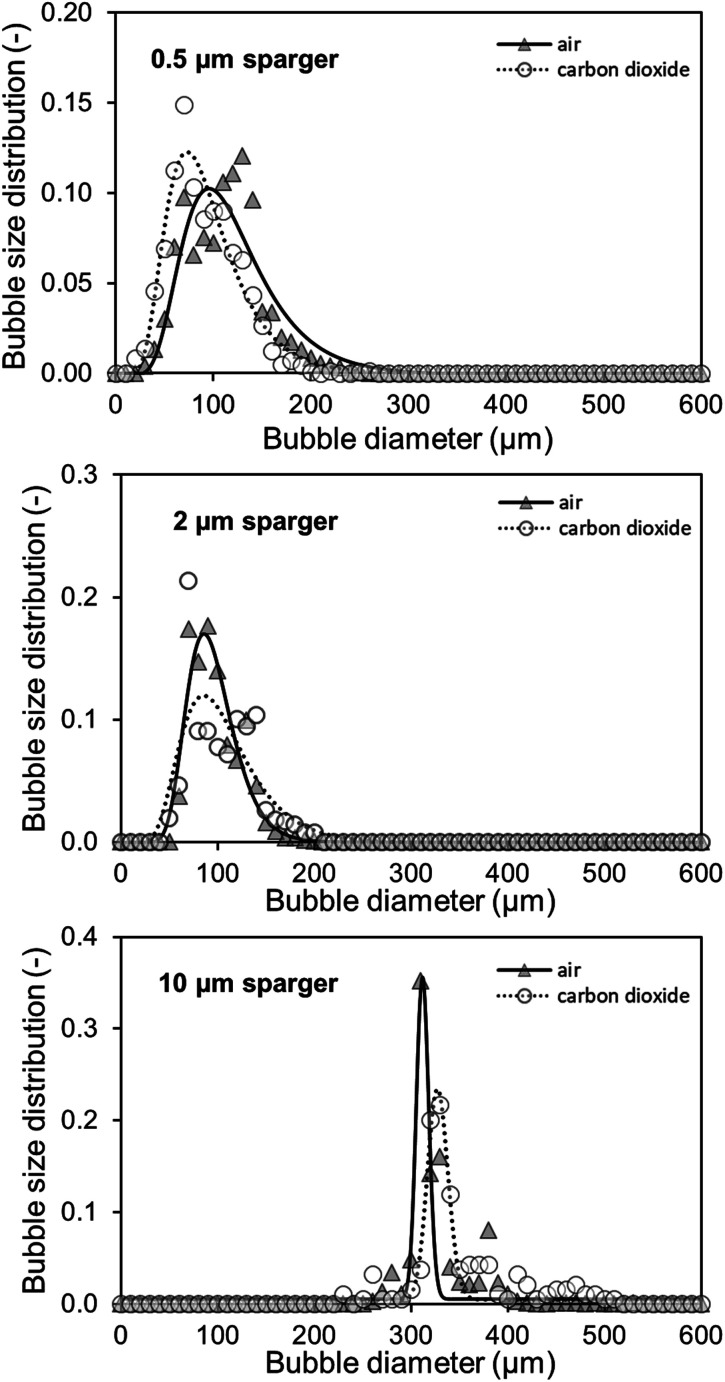
Determination of bubble size distributions for the aeration of 150 ml in 1 M triethanolamine with 20 ml min^−1^ air and CO_2_ with different spargers in a bubble column with an inner diameter of 31.9 mm at saturation under ambient conditions. The bubbles were detected with the Sopat-VI Sc probe-based microscope and analysed by the integrated image analysis software. Log-normal regressions with the following *R*^2^ for air and CO_2_ were calculated, respectively: 0.5 μm sparger: 0.88 and 0.96; 2 μm sparger: 0.93 and 0.76; 10 μm sparger: 0.80 and 0.91.

The measured BSD in Fig. 6 confirms that both small pore size spargers produce similar BSD and that using the 10 μm sparger leads to a BSD with a three times higher *d*_50_ of 314 μm. As a result, the specific interfacial area for the two spargers with the small pores is much higher, achieving in consequence an overall higher *k*_L_*a*. Performing measurements above 200 ml min^−1^ was not feasible as the column height was reached due to high gas hold-up and foaming. Furthermore, at higher gassing rates, it could no longer be differentiated if the gas bubbles were still in the liquid or already part of the foam.

As mentioned, for the calculation of the *k*_L_*a* for CO_2_, the specific surface area of air and CO_2_ bubbles needs to be known or assumed to be comparable, when using the established correlations.^31,34^ For macrobubble aeration, it is often assumed that the specific surface area is comparable for air and CO_2_.^31,32^ On the basis of achieving microbubble aeration, where the accelerated dissolution assisted by the Laplace pressure is a major driver of the high mass transfer, the comparability of air and CO_2_ microbubble aeration needs to be examined. Furthermore, the gas composition influences the interfacial tension in the system, which affects the mass transfer.^35^ Therefore, alongside the *k*_L_*a* measurements, the air and CO_2_ BSD were measured and compared at saturation level (Fig. 6). At saturation, the different solubility concentrations of both gases can be neglected to affect the bubble size as the system is in equilibrium and no mass transfer rate can be detected in the bulk.

Both the 0.5 μm sparger and the 2 μm sparger produce also similar BSD in this comparison, which supports the conclusion of similar sparger characteristics for the *k*_L_*a* measurements and BSD measurements at saturation. Furthermore, the 10 μm sparger produces much bigger bubbles compared to the other spargers. This results in smaller specific surface areas and is consistent with the measured *k*_L_*a*. The BSD for air and CO_2_ is about the same for the tested spargers, which is confirmed by performing a double-sided *t*-test. For the *t*-test, the measured cumulative distributions of air and CO_2_ with the three different spargers are merged, respectively. In this way, the range of 10–800 μm, where all bubbles are detected, was compared. The data sets consisted each of 80 data points with a bubble size resolution of 10 μm. The null hypothesis is highly significant with a *p*-value of 0.964 for there being no difference in the BSD between both gases. Therefore, it can be assumed that the specific surface area will be similar for the transfer of the *k*_L_*a* values from air oxygen to CO_2_. Additionally, the diffusion constants of air oxygen and CO_2_ in water are comparable with 2.10 × 10^−5^ cm^2^ s^−1^ and 1.92 × 10^−5^ cm^2^ s^−1^ at 25 °C, respectively.^36^ Based on these diffusion constants, the expected (*k*_L_)_CO_2__, calculated with eqn (4) is only 4.4% smaller than the (*k*_L_)_O_2__. The main difference, which affects the mass transfer for both gases are consequently their different solubilities. In water at 25 °C, oxygen and CO_2_ have Henry constants of 1.3 M atm^−1^ and 34 M atm^−1^, respectively.^37^ Therefore, a several fold higher mass transfer rate is expected for CO_2_. In aqueous TEA, the concentration difference further multiplies as even higher CO_2_ solubilities are achieved. This results in a much higher concentration gradient and thus much higher mass transfer rates.

### Mass transfer efficiency

Apart from a high mass transfer rate, mass transfer efficiencies are important to reduce waste gas and energy. For the *k*_L_*a* measurements with air, standard oxygen transfer rates (SOTR) are calculated with eqn (5), taking the saturation concentration (*c*_∞_) into account. The standard oxygen transfer efficiency (SOTE) is calculated by eqn (6) and is the ratio of the SOTR and the supplied oxygen rate (*ẇ*_O_2__). This gives the percentage of applied oxygen molecules that dissolve in the liquid volume (*V*_l_).

5 SOTR = *k*_L_*a* × *c*_∞_ × *V*_l_

6
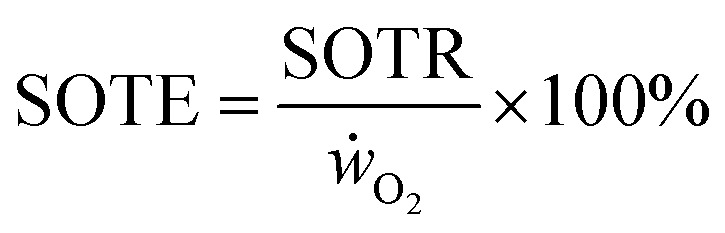


**Fig. 7:**
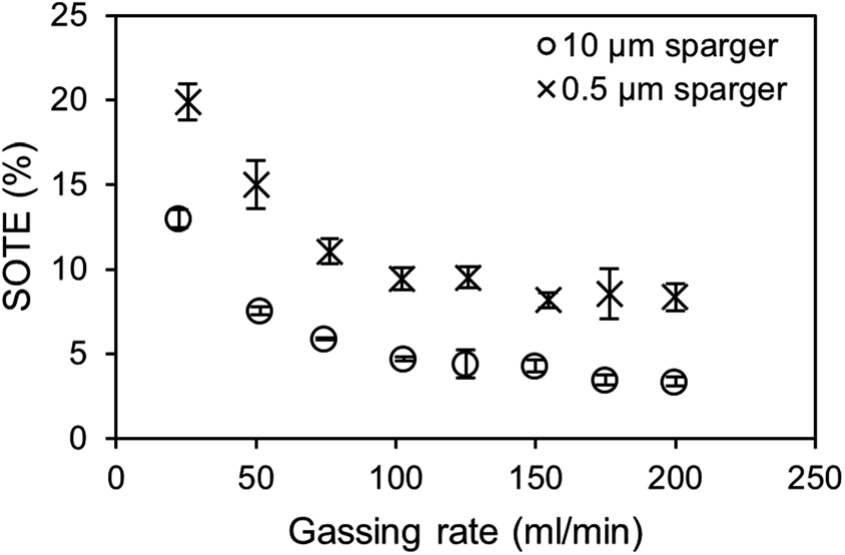
Comparison of standard oxygen transfer efficiencies (SOTE) depending on the gassing rate and sparger mean pore size. Values were calculated from the *k*_L_*a* measurements shown in Fig. 3.

In the bubble column setup, the highest SOTE are obtained at the lowest gassing rates for both the 0.5 μm and 10 μm sparger (Fig. 7). At the lowest tested gassing rate of 25 ml min^−1^, around 20% of the injected oxygen dissolves in the medium for the 0.5 μm sparger, which is around double the efficiency compared to the 10 μm sparger. It should be noted that in contrast to the *k*_L_*a* measurements, where the saturation level changes, the measured BSD at 20 ml min^−1^ air is determined at air oxygen saturation. Therefore, the effect of saturation level on BSD and *k*_L_*a* is not included in this comparison. It can be observed for both spargers in Fig. 7, that the SOTE gets independent at high gassing rates. The production of bubble sizes largely independent of the mean pore size is characteristic for the formation of a secondary bubble formation above the pores after reaching a critical gassing rate.^38^ This secondary bubble formation can basically be viewed as a coalescence driven process.

For an estimation of the standard CO_2_ mass transfer efficiency (SCTE) based on the SOTE, even higher efficiencies due to higher saturation concentrations of CO_2_ are achievable, which are further enhanced by using amines. However, the SCTE would decrease when reaching complete saturation, due to the previously described effects on the *k*_L_*a*. The incorporation of the enzymatic carboxylation of resorcinol as model reaction would reduce the loss of efficiency due to the consumption of bound CO_2_ and establishment of a steady concentration gradient. Further optimization of the aeration efficiency can be achieved by scale-up and raising the *H*/*D* ratio of the bubble column. This results in longer residence times of bubbles and an increased hydrostatic pressure, leading to an increased saturation concentration and a higher mass transfer gradient.

### Biocatalytic application

**Fig. 8:**
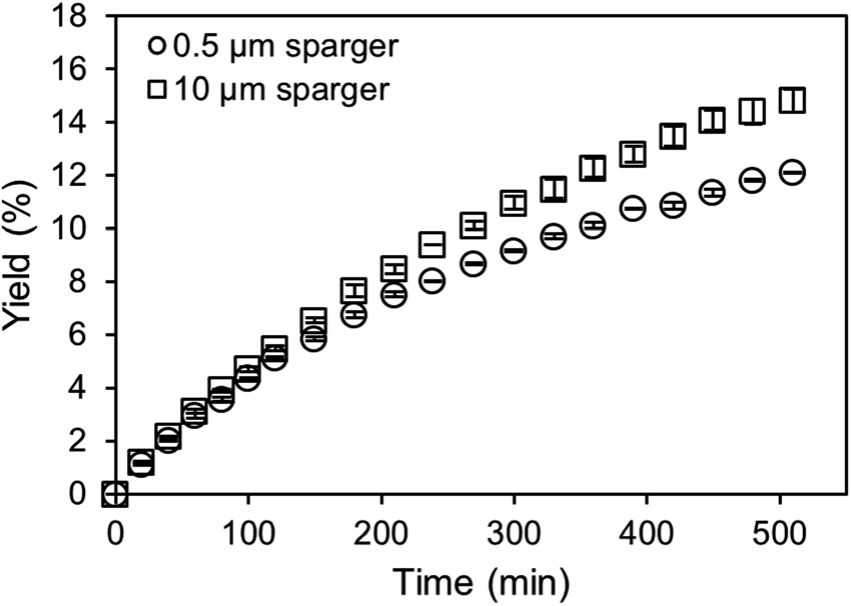
Carboxylation of 10 mM resorcinol in 1 M CO_2_-saturated triethanolamine by 12.5 μg ml^−1^ carboxylase at 30 °C aerated with 100 ml min^−1^ CO_2_ using different spargers.

**Fig. 9:**
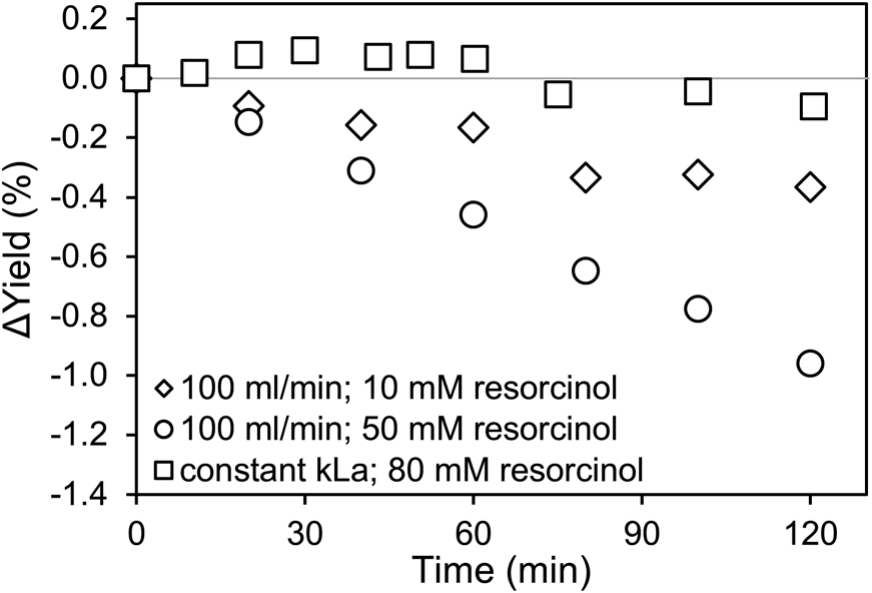
Yield comparison for different carboxylation reactions of resorcinol in 1 M CO_2_-saturated triethanolamine by 12.5 μg ml^−1^ carboxylase at 30 °C aerated with CO_2_ using a microbubble (MB) and submillibubble (SB) sparger. Δ*Y* is calculated by subtracting the achieved yield for the MB aeration with the achieved yield, when using the SB sparger.

For the application of amines in biocatalytic carboxylation, the critical step is to prevent pH dependent enzyme deactivation. Therefore, neutralization of the highly alkaline amine solution by CO_2_ saturation prior to addition of the biocatalyst is mandatory. After initial presaturation, concentration changes of the dissolved CO_2_ are tied to the enzymatic carboxylation, where the dissolved CO_2_ is consumed. An active CO_2_ aeration guarantees a constant high level of dissolved CO_2_, which improves the yield. Consequently, a highly efficient aeration with minimal waste gas stream is preferred. Both, the 0.5 μm microbubble sparger and the 10 μm submillibubble sparger are compared at the same gassing rate as well as same applied mass transfer rates (Fig. 8 and 9). At 10 mM starting concentration of the substrate resorcinol, the utilization of the 10 μm sparger achieves a higher productivity compared at 100 ml CO_2_ per minute with minimal deviation between biological duplicates. One possibility for the different productivities is enzyme deactivation during the course of the reaction. The most likely reason is enzyme deactivation due to a difference in the gas–liquid interface caused by utilization of different spargers. In general, enzymes are prone to deactivate at gas–liquid interfaces as demonstrated for several different enzymes.^39,40^ Different effects, such as a mass transfer limitation can be excluded as the reaction is much slower than the applied CO_2_ mass transfer rate, which is also confirmed by a constant pH during the entire reaction. For the same reason of having a very slow reaction and constant pH, differences in mixing behavior due to different bubble sizes and therefore effected rising velocities and impulses are assumed to be neglectable.

For the utilization of the 10 μm sparger, the presumed deactivation results in over 20% higher yields after 5 hours. The yield difference is even more pronounced when higher initial substrate concentrations are applied (Fig. 9). Higher substrate concentrations increase the reaction rate due to enzyme kinetics, which results in higher overall productivities. Therefore, the reaction could be more sensitive to deactivating influences in the compared time up to 120 min. In contrast to the measurements at identical gassing rates, no yield difference is observed when performing the reaction at comparable applied *k*_L_*a* of approximately 140 h^−1^ for both cases even at a higher substrate concentration of 80 mM.

Microbubbles have a lower coalescence tendency, which also results in higher foam stability compared to macrobubbles. Additionally, proteins are known to accumulate in foam, which can be used as application for protein recovery.^41^ Yet, the flotation of enzymes would cause a reduced observed activity. Comparing microbubble and macrobubble aeration at similar *k*_L_*a*, no yield difference is observed, which is generally expected if there is no mass transfer limitation. Connecting this result with the experiments for different gassing rates, where also no mass transfer limitation existed, variation in the amount of gas–liquid interface is the probable cause for the deviating productivities. Performing the biotransformation at comparable *k*_L_*a*, the surface area is likely to be comparable. For a better comparison, the measured BSD and Sauter mean diameter (*d*_32_) are used to calculate the rate of interfacial area generation (*ȧ*) using the specific volume (SV) for spheres^42^ and gassing rate (*V̇*):7
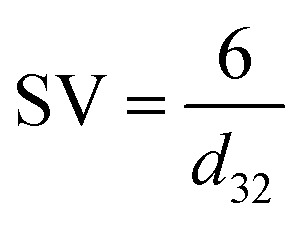

8 *ȧ* = SV × *V̇*

Comparing the calculated *ȧ* for the determined Sauter mean diameter (Table 2), the microbubble aerator generates a 2.5 times higher interfacial area per minute. This supports the theory that an increased gas–liquid interfacial area is the reason for enzyme deactivation resulting in the observed differences in productivities. At comparable *k*_L_*a*, both spargers generate a similar interfacial area per minute, which only differed by 30% explaining the similar productivities. It should be noted that the calculation is only based on the *d*_32_ for an aeration at 20 ml min^−1^. Combined with the BSD measurements and lognormal distribution fittings of the BSD with *R*^2^ in some cases below 0.8, the calculated values are in good agreement with the experimental data from the biotransformation. The presumed deactivation behavior, expected to be mainly caused by the interfacial area, is already under further investigation. Additional factors and interdependencies could also affect enzyme deactivation, such as shear forces introduced by bubble bursting and coalescence, foam formation and processes at the interfacial area as well as the unique shrinking behavior of microbubbles.^13^

**Table 2:** Comparison of the rate of the surface area generation *ȧ* based on the measured Sauter mean diameter *d*_32_ dependent on the gassing rate *V̇*

	Sparger	*d*_32_ (μm)	*V̇* (ml min^−1^)	*ȧ* (m^2^ min^−1^)
Comparable gassing rate	0.5 μm	141	100	4.26
	10 μm	354	100	1.70
Comparable *k*_L_*a*	0.5 μm	112^a^	50	2.67
	10 μm	349	200	3.44

a Sauter mean diameter value is based on the measurement at 20 ml min^−1^ as the BSD was not measured at 50 ml min^−1^.

## Conclusions

The enzymatic carboxylation of resorcinol in aqueous triethanolamine, acting as amine scrubber, enables the fixation of CO_2_ and simultaneous generation of valuable 2,6-dihydroxybenzoic acid. Microbubble aeration is shown to represent a suitable technique to generate high *k*_L_*a* values above 300 h^−1^ for a fast and efficient aeration, reducing the amount of gas wasted. Especially, the CO_2_ saturation level is found to highly affect the BSD in the amine–CO_2_ system, enabling the generation of much smaller bubbles at low CO_2_ saturations. The results indicate that the enhancing effect of the accelerated dissolution assisted by the Laplace pressure of microbubbles and coalescence at saturation cause the shifting of bubble diameter in the BSDs. Furthermore, it was found that the BSD is independent of the investigated gases, air and CO_2_, at saturation. For the application of the amine–CO_2_ system for enzymatic carboxylation, the generation of interfacial area was found to correlate with loss of enzyme activity the during reaction. Therefore, the rate of surface generation, which is dependent on the resulting BSD and applied gassing rate needs to be minimized while still achieving high *k*_L_*a* values. Microbubble aeration is shown to provide these requirements more efficiently compared to submillibubble aeration. In the case of microbubbles, the provided higher specific surface area enables the reduction of the gassing rate, preventing higher rates of surface generation and foaming under the investigated conditions. The provided insights into the amine–CO_2_ system could enable a more comprehensive understanding and the development of an industrial process based on utilization of CO_2_ in the enzymatic carboxylation.

## Conflicts of interest

There are no conflicts to declare.

## Supplementary Material
